# Relating retinal blood flow and vessel morphology in sickle cell retinopathy

**DOI:** 10.1038/s41433-019-0604-y

**Published:** 2019-09-26

**Authors:** Jennifer Cano, Shayan Farzad, Maziyar M. Khansari, Ou Tan, David Huang, Jennifer I. Lim, Mahnaz Shahidi

**Affiliations:** 1 Ophthalmology, University of Southern California, Los Angeles, CA United States; 2 Casey Eye Institute, Oregon Health & Science University, Portland, OR United States; 3 Ophthalmology and Visual Sciences, University of Illinois at Chicago, Chicago, IL United States

## Abstract

**Purpose:**

The purpose of the current study was to determine associations between retinal blood flow and vessel morphology metrics in sickle cell retinopathy (SCR) and healthy normal control (NC) subjects.

**Methods:**

Optical coherence tomography angiography (OCTA) and Doppler OCT imaging were performed in 12 SCR (15 eyes) and 19 NC (26 eyes) subjects. Vessel tortuosity was measured using a dedicated image analysis algorithm applied to OCTA images. Vessel density and spacing between vessels were determined from OCTA images by a fractal dimension analysis method. Retinal blood flow was quantified using a phase-resolved technique applied to *en face* Doppler OCT images.

**Results:**

There was a significant association between increased retinal blood flow and increased vessel tortuosity (*P* = 0.03). Furthermore, increased retinal blood flow was associated with increased vessel density (*P* = 0.03) and decreased spacing between small vessels (*P* = 0.01). There was no significant association between retinal blood flow and spacing between large vessels (*P* = 0.11). Vessel tortuosity and blood flow were increased, whereas spacing between small vessels was decreased in SCR compared to NC group (*P* ≤ 0.03). There were no significant differences in vessel density or spacing between large vessels between the SCR and NC groups (*P* ≥ 0.31).

**Conclusions:**

Associations between retinal hemodynamics and vessel morphology were reported, providing better understanding of retinal pathophysiology and insight into potential quantitative biomarkers to evaluate SCR.

## Introduction

Vaso-occlusion caused by sickling of erythrocytes in sickle cell disease (SCD) occurs in all tissues throughout the body, including retinal tissue [1]. Sickle cell retinopathy (SCR) is a vision-threatening eye disease caused by retinal ischemia triggered by vaso-occlusions [2]. Although homozygous SCD (HbSS) is the most severe systemic form of the disease, proliferative SCR occurs in individuals with less severe systemic disease, namely heterozygous SCD (HbSC) and sickle thalassemia (HbSThal) [3, 4]. Vascular pathologies associated with proliferative SCR include the following stages of peripheral arterial occlusion, arteriovenous anastomoses, peripheral neovascularization, vitreous haemorrhage, and retinal detachment [5]. Complications of advanced SCR can lead to vision loss, hence the importance of screening and disease management [6].

Diagnosis of stages of SCR is primarily based on visualization of retinal vasculopathies [7]. Recently, optical coherence tomography angiography (OCTA) has gained recognition through its increased sensitivity in diagnosing SCR, compared to fluorescein angiography (FA) [8]. It is capable of providing high-resolution images of the retinal capillary plexus, which allows for non-invasive quantitative assessment of microvascular perfusion [9]. Alterations in retinal vessel morphology due to SCR, namely decreased retinal vessel density [10, 11], enlarged foveal avascular zone (FAZ) [10, 12], and an association between decreased vessel density and increased FAZ [13] have been reported using OCTA imaging. Changes in the capillary density are thought to be a result of micro-occlusions and consequent capillary dropout caused by sickled cells [14]. Additionally, decreased vessel density has been detected in areas of retinal thinning, which is presumably caused by ischemia [10, 11]. Vessel tortuosity is an important biomarker because it can be present in early stages of SCR, therefore, prompting early monitoring [15]. Increases in retinal vessel tortuosity have also been identified in patients with SCR [11, 16]. Another observed characteristic of SCR is increased total retinal blood flow (TRBF), which may be a compensatory mechanism for insufficient tissue oxygenation [17, 18]. However, it is not known whether there is an association between changes in retinal vessel morphology and haemodynamics. The purpose of the current study was to test the hypothesis that TRBF is linearly related to vessel tortuosity and density.

## Methods

### Subjects

The study was conducted at the University of Illinois at Chicago and University of Southern California and was approved by their corresponding Institutional Review Boards. Informed consents were obtained in accordance to the tenets of Declaration of Helsinki, after the study was explained to the subjects. Subjects were categorized into SCR or healthy normal control (NC) groups based on clinical history and ocular examination. Twelve SCR subjects (two males and 10 females) and 19 NC subjects (10 males and nine females) enrolled in the study. Among the SCR group, 6, 4, and 2 subjects had HbSS, HbSC, and HbSThal genotypes, respectively.

Prior to imaging, subjects’ eyes were anesthetized using proparacaine hydrochloride 0.5% (Bausch & Lomb), then dilated using tropicamide 1.0% (Alcon Laboratories, Inc.) and phenylephrine hydrochloride 2.5% (Paragon BioTek Inc.). Haematocrit was measured from a blood sample obtained from a finger-prick and centrifuged in a micro-haematocrit centrifuge (Unico, Dayton, NJ). Blood pressure (BP) was recorded using a wrist cuff to obtain the mean arterial pressure (MAP) = 1/3(systolic BP) + 2/3(diastolic BP). Three MAP measurements were averaged.

Data were not available in both eyes of all subjects because either only one eye was imaged, or image quality was not adequate. Images were available in 15 SCR eyes (six right eyes (OD) and nine left eyes (OS)), and 26 NC eyes (10 OD and 16 OS). SCR stage was determined based on the Goldberg classification [5] by clinical evaluation of a retina specialist (JIL). Two, 9, and 4 eyes had SCR stage 0, II, and III, respectively.

### Image acquisition

Imaging was performed using a commercially available Avanti OCTA system (Optovue, Inc., Fremont, CA). OCTA images of the superficial capillary plexus were acquired in a 6 × 6 mm retinal region, centered on the fovea. The superficial retinal vascular layer was defined by the Optovue software within the nerve fibre and ganglion cell layers. One OCTA image per eye with scan quality score greater than 3, as evaluated by the instrument’s software, was selected for analysis. *En face* Doppler OCT images were generated using a custom scan protocol for imaging a 2 × 2 mm area centered on the optic nerve head (ONH). The laser wavelength was 840 ± 45 nm with an axial scan rate of 70 KHz and had an axial scan (A-scan) depth resolution of 5 μm. The volume scan contained 80 B-scans, and each B-scan contained 500 A-scans. Five or more volume scans were acquired per eye.

### Image analysis

#### Vessel tortuosity

Tortuosity of retinal vessels was quantified by our previously validated vessel tortuosity index (VTI) using 6 × 6 mm OCTA images of the retinal superficial capillary plexus [16]. Retinal vessels were detected using a k-means clustering algorithm to provide a binary vessel map. Centrelines between the bifurcation points were extracted using distance transformation by selection of vessel endpoints on the binary vessel map. The mathematical derivation of VTI is given by the following equation: $VTI = \frac{{0.1SD_\theta .N.M.L_A}}{{L_C}}$, where SD_θ_ represents the local angle change, M is the amplitude of the curvature along the vessel centreline, N is the number of critical points, L_A_ is the centreline length, and L_C_ is the centreline chord length. VTI was calculated for each of the extracted centrelines and averaged per eye. The minimum value for VTI is theoretically zero, which corresponds to a straight line, whereas there is no theoretical maximum value for VTI.

#### Vessel density

**Fig. 1:**
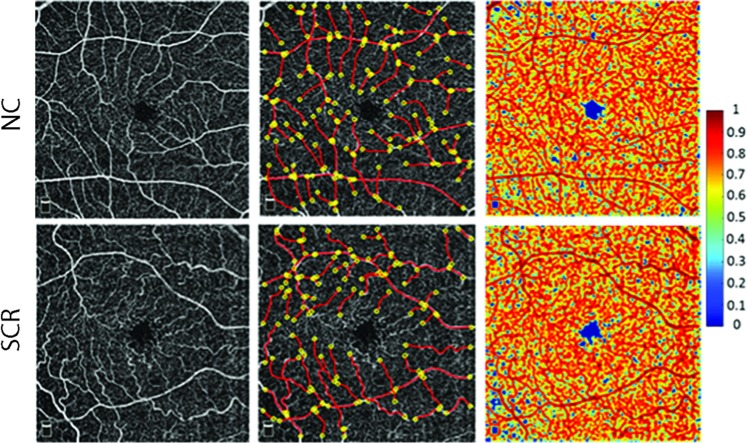
(Left) OCTA images in a 6 × 6 mm retinal superficial capillary plexus acquired in healthy control (NC) (top) and sickle cell retinopathy (SCR) subjects (bottom). (Middle) Vessel endpoints (yellow circles) and centrelines (red lines) used for vessel tortuosity measurements are overlaid on OCTA images in the same NC and SCR subjects. (Right) Fractal dimension ratio (FDR) images in the same NC and SCR subjects. The colour bar shows FDR values ranging from 0 to 1

Retinal vessel density was assessed based on a previously established local fractal dimension (LFD) analysis method applied to OCTA images [19, 20]. A moving window size of 3 × 3 pixels was used to calculate the LFD of each pixel which varied with the distribution of vessels around it. The FD ratio (FDR) was calculated as the ratio of LFD of each pixel to the maximum LFD and provided a graphic representation of the probability index of presence of vessel of a certain size at each pixel. As previously described [19], FDR between 0.7 and 1 corresponded to large and small vessels, between 0.3 and 0.7 corresponded to spacings between small vessels, and less than 0.3 corresponded to spacings between large vessels. LFD metrics, namely, vessel density (VD), spacing between small vessels (SSV), and spacing between large vessels (SLV) were calculated as the ratio of number of pixels to the total number of pixels in the image with 0.7 ≤ FDR ≤ 1, 0.3 < FDR < 0.7, and 0 ≤ FDR ≤ 0.3, respectively. LFD metrics values of 0 and 1 indicate 0% and 100% of the total image, respectively. Examples of OCTA images in NC and SCR subjects, extracted centrelines for tortuosity analysis, and their respective FDR images generated by fractal analysis are shown in Fig. 1.

#### Blood flow

**Fig. 2:**
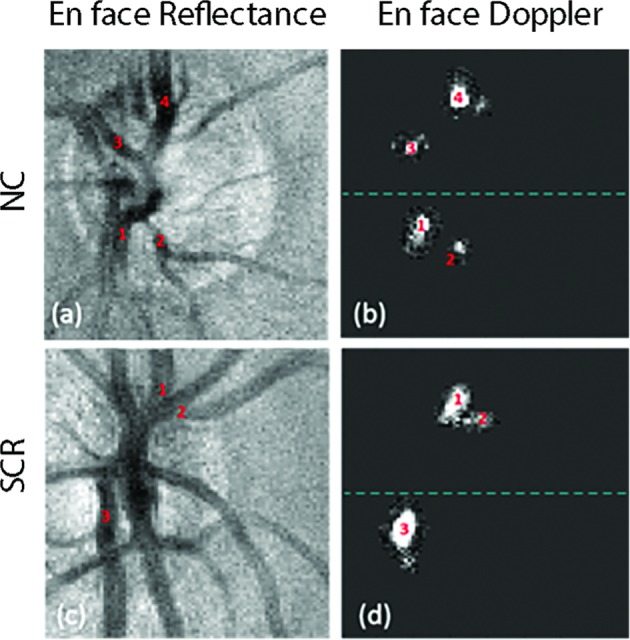
**a** *En face* reflectance image of the optic nerve head acquired in a healthy control (NC) subject. **b** *En face* Doppler image displaying detected retinal veins in the same NC subject. **c, d** *En face* reflectance and Doppler images acquired in a sickle cell retinopathy (SCR) subject

A previously published image analysis software was employed to measure TRBF from Doppler OCT images [21]. A phase-resolved technique was applied to measure Doppler phase shift. Blood flow within retinal veins was calculated on each *en face* plane. For each vein, the highest flow measurement among *en face* planes was recorded. TRBF was measured based on summation of retinal vein flow measurements in each volume and averaged over the volumes. TRBF was measured based on summation of retinal vein flow measurements in each volume and averaged over all volumes, ranging between 3 and 26. Examples of *en face* OCT images of the ONH and vein segments in NC and SCR subjects are shown in Fig. 2.

### Statistical analysis

Prior to statistical analyses, normality of data distribution was confirmed using Shapiro-Wilk tests and graphical visualization of quantile-quantile plots. To compare age, MAP, and haematocrit between SCR and NC groups, unpaired t-tests were used and equality of variances were confirmed by folded F-tests. Sex and race were compared using Fisher’s exact test. A variance components, mixed model analysis was conducted to evaluate the association between TRBF (independent variable) and VTI and LFD metrics (dependent variables). With our sample size of 41, the study had 80% power to detect a correlation coefficient of 0.43 or higher at the alpha level of 0.05. Mixed model analysis was also used to compare VTI, LFD metrics, and TRBF between SCR and NC groups. Disease group was modelled as a fixed, independent variable, while VTI, LFD metrics, and TRBF were each modelled as dependent variables. Models adjusted for eye (accounting for intrasubject eye correlations) and MAP, and subjects were modelled as random effects. Statistical tests were 2-sided and significance was accepted at P ≤ 0.05. Analyses were performed using SAS software (SAS, version 9.4; SAS Institute Inc., Cary, NC).

## Results

Subject demographic and clinical characteristics are shown in Table 1. The mean age in the SCR and NC groups were 44 ± 14 years (mean ± standard deviation) and 36 ± 15 years, respectively. Age and sex were not significantly different between SCR and NC groups (*P* ≥ 0.07); however, race, MAP, and haematocrit did differ between groups (*P* ≤ 0.002).

**Table 1:** Subjects’ demographics and clinical characteristics of SCR and NC groups

	SCR (*n* = 12)	NC (*n* = 19)	*P*-value
Sex			
Male	2	10	0.07
Female	10	9	
Race			
White	0	8	<0.0001
African-American	12	2	
Hispanic/Latino	0	9	
Age (years)	44 ± 14	36 ± 15	0.19
MAP (mmHg)	86 ± 13	102 ± 13	0.002
Haematocrit (%)	29 ± 4	44 ± 3	<0.0001

Age, MAP, and haematocrit are presented as mean ± standard deviation. Sex and race were compared using Fisher’s exact tests. Age, MAP, and haematocrit were compared by unpaired *t*-tests

*SCR*   sickle cell retinopathy, *NC*  normal control, *MAP*  mean arterial pressure

Estimates represent changes in indices per 100 uL/min of TRBF. There was a significant association between increased TRBF and increased VTI (β = 0.49; 95% CI: 0.07, 0.92; *P* = 0.03). Similarly, increased TRBF was associated with increased VD (β = 0.10; 95% CI: 0.02, 0.17; *P* = 0.03). TRBF and SSV were inversely associated (β = -0.05; 95% CI: −0.08, −0.02; *P* = 0.01), such that increased TRBF was related to decreased SSV. There was no significant association between TRBF and SLV (β = −0.05; 95% CI: −0.11, 0.02; P ≥ 0.11).

Mean, standard deviation, and effect estimates of VTI, LFD metrics and TRBF in SCR and NC groups are shown in Table 2. VTI was increased in SCR compared to NC group (β = 0.28; 95% CI: 0.05, 0.51; *P* = 0.02). SSV was decreased in SCR compared to NC group (β = −0.03; 95% CI: −0.04, −0.02; *P* = 0.002). There were no significant differences in VD or SLV between the SCR and NC groups (*P* ≥ 0.31). TRBF was increased in SCR compared to NC group (β = 26.27; 95% CI: 3.58, 48.97; *P* = 0.03).

**Table 2:** Mean VTI, LFD metrics, and TRBF in SCR and NC groups

	SCR (15 eyes)	NC (26 eyes)	β	*P*-value
VTI	0.61 ± 0.36	0.42 ± 0.11	0.28	0.02*
VD	0.51 ± 0.06	0.50 ± 0.04	0.01	0.47
SSV	0.32 ± 0.02	0.35 ± 0.01	−0.03	0.002*
SLV	0.17 ± 0.05	0.15 ± 0.03	0.02	0.31
TRBF (μL/min)	70.42 ± 32.30	45.85 ± 11.97	26.27	0.03*

Data are presented as mean ± standard deviation; β = effect estimates were derived by mixed model analysis, adjusted for eye and mean arterial pressure. Effect estimates are reported with NC as reference. Statistically significant difference is indicated by an asterisk (*)

*SCR*   sickle cell retinopathy, *NC*  normal control, *VTI*  vessel tortuosity index, *VD*  vessel density, *SSV*   spacing between small vessels, *SLV*  spacing between large vessels, *TRBF*  total retinal blood flow

## Discussion

Detection of retinal vasculopathies in individuals with SCR is crucial for monitoring disease progression and development of vision-threatening complications. Imaging modalities, such as OCTA, provide useful quantitative measures to assess retinal vasculopathies for better understanding of the microvascular manifestations of SCR [22]. Previous studies have found increased vessel tortuosity [11, 16], decreased vessel density [10, 11], and increased blood flow [17, 18] in SCR subjects. In the current study, associations between retinal haemodynamics and vessel morphology metrics were reported.

Vessel tortuosity is thought to be a compensatory restructuring of vessels in response to haemodynamics alterations [23]. Retinal vessels are thought to become tortuous due to increased blood flow resulting from reduced capillary resistance subsequent to arteriovenous shunting [24–26]. Our finding of an association between increased TRBF and increased VTI is consistent with reported results in animal models of common carotid artery (CCA) occlusion. In one study, induced tortuosity in the basilar artery following a significant increase in blood flow was demonstrated in rabbits that underwent bilateral CCA ligation [27]. Furthermore, vascular remodelling of tortuosity of the left CCA was present following increased blood flow in a rabbit arteriovenous fistula model [23].

Regulation of blood flow and changes in vessel density are dependent on changes in blood oxygen concentrations [28]. In the current study, increased TRBF was associated with increased VD, which may suggest overcompensation for inadequate retinal tissue oxygenation. The results suggest that hyperperfusion ensuing a rise in TRBF is driving the observed increase in vessel perfusion. Due to the novelty of this topic, previous research is not available to corroborate this. However, a positive correlation between capillary blood flow and density has been reported in cerebral regions of rats subsequent to periods of ischemia [29, 30]. Interestingly, we found an inverse association between TRBF and SSV, such that increased TRBF was related to decreased spacing between small vessels. One plausible explanation for this result is an increase in vessel diameters or vasodilation. Our finding is in agreement with previous studies that showed vasodilation was induced by hypoxic conditions due to sickling [31] and increased endothelial shear stress [32], simultaneously increasing blood flow.

Vessel tortuosity has long been identified as a feature of SCR, and indeed is used in its staging [5, 33, 34]. Our finding of increased VTI in SCR is consistent with previously published studies [11, 16]. However, we did not find a significant decrease in VD in SCR, as others have reported [10, 11]. It has been suggested that vessel density is decreased in subjects with proliferative retinopathy, compared to other stages [35]. The differences in findings between studies may be attributed to variations in stage of SCR, disease genotype, demographics of subjects, sample size and methodologies. Nevertheless, the finding of reduced SSV in our study was also observed by Alam and associates, as they reported decreased spacing between small vessels in SCR subjects [13]. They suggested in order to offset an increase in non-vascular regions, spacing between small vessels are decreased. Finally, we showed increased TRBF in SCR consistent with published literature [17, 18]. It is presumed blood flow in individuals with SCR is increased to counter anaemic conditions and low haematocrit levels [17].

Our study was limited by a small sample size, which did not permit us to divide subjects by SCR stage and investigate differences between stages. Another limitation was the inability to match races between the SCR and NC subjects, although a literature search of racial differences in retinal blood flow, density, or tortuosity did not reveal any differences. Finally, although associations between retinal haemodynamics and vessel morphology were reported, their temporal relationship sequences, or whether both occur in parallel due to the underlying disease could not be elucidated, requiring a longitudinal study to investigate causality. Nevertheless, the current study demonstrated a relationship between retinal blood flow and vessel morphology metrics. Future studies in a larger cohort with addition of measurements of vascular oxygenation are warranted to better characterize and advance our understanding of SCR pathophysiology.

In summary, this is the first study to report associations between increased retinal blood flow and abnormalities in vessel morphology. The findings contribute to the understanding of retinal pathophysiology and may provide insight into potential quantitative biomarkers to evaluate SCR.

## Summary

### What was known before

- Previous literature has reported increased retinal vessel tortuosity, decreased vessel density, and increased blood flow in SCR subjects.

### What this study adds

- This is the first study to report associations between increased retinal blood flow and abnormalities in vessel morphology.
- It contributes to the understanding of retinal pathophysiology and provides insight into potential quantitative biomarkers to evaluate sickle cell retinopathy.
